# Comparison of lung cancer occurring in fibrotic versus non-fibrotic lung on chest CT

**DOI:** 10.1186/s12967-023-04645-y

**Published:** 2024-01-16

**Authors:** Mary M. Salvatore, Yucheng Liu, Boyu Peng, Hao Yun Hsu, Anjali Saqi, Wei-Yann Tsai, Cheng-Shiun Leu, Sachin Jambawalikar

**Affiliations:** 1 Department of Radiology, Columbia University Irving Medical Center, 622 W 168th Street, New York, NY 10032 USA; 2 Department of Pathology, Columbia University Irving Medical Center, New York, NY USA; 3 Department of Biostatistics, Columbia University, New York, NY USA

**Keywords:** Pulmonary fibrosis, Lung cancer, Pulmonary nodules, Doubling time, Lung cancer screening

## Abstract

**Purpose:**

Evaluate the behavior of lung nodules occurring in areas of pulmonary fibrosis and compare them to pulmonary nodules occurring in the non-fibrotic lung parenchyma.

**Methods:**

This retrospective review of chest CT scans and electronic medical records received expedited IRB approval and a waiver of informed consent. 4500 consecutive patients with a chest CT scan report containing the word fibrosis or a specific type of fibrosis were identified using the system M*Model Catalyst (Maplewood, Minnesota, U.S.). The largest nodule was measured in the longest dimension and re-evaluated, in the same way, on the follow-up exam if multiple time points were available. The nodule doubling time was calculated. If the patient developed cancer, the histologic diagnosis was documented.

**Results:**

Six hundred and nine patients were found to have at least one pulmonary nodule on either the first or the second CT scan. 274 of the largest pulmonary nodules were in the fibrotic tissue and 335 were in the non-fibrotic lung parenchyma. Pathology proven cancer was more common in nodules occurring in areas of pulmonary fibrosis compared to nodules occurring in areas of non-fibrotic lung (34% vs 15%, p < 0.01). Adenocarcinoma was the most common cell type in both groups but more frequent in cancers occurring in non-fibrotic tissue. In the non-fibrotic lung, 1 of 126 (0.8%) of nodules measuring 1 to 6 mm were cancer. In contrast, 5 of 49 (10.2%) of nodules in fibrosis measuring 1 to 6 mm represented biopsy-proven cancer (p < 0.01). The doubling time for squamous cell cancer was shorter in the fibrotic lung compared to non-fibrotic lung, however, the difference was not statistically significant (p = 0.24). 15 incident lung nodules on second CT obtained ≤ 18 months after first CT scan was found in fibrotic lung and eight (53%) were diagnosed as cancer.

**Conclusions:**

Nodules occurring in fibrotic lung tissue are more likely to be cancer than nodules in the nonfibrotic lung. Incident pulmonary nodules in pulmonary fibrosis have a high likelihood of being cancer.

## Introduction

Lung cancer screening has decreased lung cancer mortality and all-cause mortality because of earlier diagnosis and treatment [1, 2]. Henschke et al. found that 23% of 1000 patients who had a 10-year smoking history had at least one lung nodule on baseline chest CT and 27 had cancer [3]. Cancers found on initial screening were slower growing than cancers found on follow-up imaging; the mean volume doubling time for non-small cell lung cancers identified on follow-up screening was 154 days, 50% of patients had adenocarcinoma, and 9% squamous cell cancer [4]. Defining the optimal follow-up interval for pulmonary nodules identified on lung cancer screening chest CT has been key to its success [4, 5].

Patients with pulmonary fibrosis are at increased risk of developing lung cancer [6–9]. Even the earliest changes of fibrosis increase a person’s risk [10]. Lung cancers that occur in pulmonary fibrosis are different from cancer occurring in normal or emphysematous lung based on their distribution and cell type with more frequent, peripheral, faster-growing squamous cell cancers in fibrosis [8]. The microenvironment of fibrosis promotes tumor growth with increased fibroblast foci and tumor-associated macrophages (M2). M2 macrophages are primarily tumor-infiltrating immune cells associated with the promotion of cancer cell growth, invasion, metastasis, and angiogenesis [11, 12].

The United States Preventative task force has recently updated its lung cancer screening guidelines to include current smokers aged 50–80 years with at least a 20-pack year history of smoking and those who quit less than 15 years ago [13]. The change was prompted by the observation that many of those diagnosed with lung cancer would not qualify for screening based on original, more rigid, screening criteria [14]. Pulmonary fibrosis diagnosis alone does not qualify a patient to participate in a lung cancer screening regimen [15], yet many fibrosis patients have repeat CT scans to follow the progression of their disease [16]. The purpose of our research was to evaluate the behavior of lung nodules in fibrosis and determine if current lung cancer screening regimens could benefit high lung cancer risk pulmonary fibrosis patients.

## Methods

This retrospective review of chest CT scans and electronic medical records from a single academic medical center in New York City received expedited internal review board approval and a waiver of informed consent. 4500 consecutive patients with a chest CT scan report containing the word fibrosis or a specific type of fibrosis were identified using the system M*Model Catalyst (Maplewood, Minnesota, U.S.). Patients were excluded if they were less than 21 years old at the time of the initial CT scan or if the images were not retrievable. If the patient had a pathology diagnosis of cancer in the lung, two CT scans prior to treatment were reviewed. If the patient had no known lung cancer diagnosis, the two most recent chest CT scans were reviewed. Due to the retrospective nature of this study, CT scans from a variety of manufacturers with variable dose and slice thickness were included. The maximum slice thickness for a minority of patients was 5 mm. Images were reviewed using standard lung window settings (W: 1500 L: − 600). The location and size of the largest solid nodule in the area of fibrotic lung or non-fibrotic lung were documented by MS with 20 years of experience. The largest nodule was measured in the longest dimension [17] and re-evaluated, in the same way, on the follow-up exam if multiple time points were available. The nodule doubling time was calculated [18, 19]. If the patient developed cancer, the type of biopsy performed and the histologic diagnosis was documented. A nodule was considered to arise in fibrosis if it was inseparable from the fibrosis, it did not have to be surrounded in its entirety by fibrotic lung tissue. In a similar manner, a nodule was considered to arise in non-fibrotic tissue if it was not continuous with the fibrosis.

The patient’s gender and age at the time of the first CT scan were recorded. When a fibrotic pattern was diagnosed (e.g. in presence of traction bronchiectasis, honeycombing, fibrotic reticulations), the radiology pattern of fibrosis was documented by a senior radiologist with over 20 years of experience. Usual interstitial pneumonia (UIP) and probable UIP were defined as subpleural, basilar predominant fibrosis with or without honeycombing on chest CT. Airway-centered fibrosis (ACF) was defined as fibrosis that surrounded the airways with or without mosaic attenuation, Nonspecific interstitial pneumonitis (NSIP) was defined as homogeneous lower lobe predominant fibrosis. Sarcoidosis was diagnosed if upper lobe posterior predominant pulmonary fibrosis. Please refer to Table 1 for a complete description of the terms. If fibrosis was not present the CT scan was described as normal or the pattern of non-fibrotic lung disease was recorded.

**Table 1:** Patterns in fibrotic and non-fibrotic lung

Fibrosis (3377)	Non-fibrotic (1057)
*Airway centered fibrosis (952)* Fibrosis along the bronchovascular bundles with mosaic attenuation	*No fibrosis (555)*
*Usual interstitial pneumonitis (697)* Subpleural basilar predominant fibrosis with or without honeycombing	*Bilateral transplant (142)*
*Sarcoid (516)* Upper lobe posterior predominant airway centered fibrosis	*Mycobacterial avium intracellular (81)* Right middle lobe and lingula predominant mucoid impaction
*Nonspecific interstitial pneumonitis (476)* Homogeneous lower lung predominant fibrosis	*Pneumonia (72)*
*Unilateral transplant (244)* Nonspecific pattern of fibrosis in the native lung with volume loss	*Bronchial disease (63)* Bronchial wall thickening and mosaic attenuation
*Combined fibrosis and emphysema (113)* Fibrosis with 10%greater centrilobular type emphysema	*Emphysema (56)* Ill-defined lucent regions of the lung measuring < 950 HU
*UIP associated with CTD (109)* UIP pattern with superimposed ground glass or consolidation	*Pulmonary artery hypertension (34*) Pulmonary artery greater than 33 mm in transverse dimension or PA/aorta ratio > 1
*Organizing pneumonia (79)* Round opacities in a peripheral or bronchovascular distribution	*Pulmonary edema (25)* Smooth interlobular septal thickening and ground glass with effusion
*Lymphocytic interstitial pneumonitis (39)* Lower lobe cysts in a bronchovascular distribution	*Effusion (13)*
*Radiation (24)* Fibrosis with well-defined margins on sagittal view	*Round atelectasis (4)* Round opacity next to a pleural abnormality
*Pleural parenchymal fibroelastosis (20)* Excessive apical pleural thickening with air bronchograms that extends along lateral pleura	*Pulmonary alveolar proteinosis (4)* Crazy paving pattern
*Cystic fibrosis (17)* Upper lung predominant cystic and or varicoid type bronchiectasis	*Lymphangitic (4)* Nodular thickening of the interlobular septa in a patient with known cancer
*Desquamative interstitial pneumonitis (17)* Homogeneous lower lobe ground-glass opacities with emphysematous type changes	*Blood (2)* Ground glass opacity with appropriate clinical history
*Respiratory bronchiolitis ILD (16)* Upper lung centrilobular nodules	*Amyloid (2)* Coarse Calcified and non-calcified pulmonary nodules
*Other (58)* Osteophyte induced fibrosis (15), Vasculitis (9), Unclassifiable (8), Asbestosis (8), Drug reaction (5), Lupus (5), Lymphangioleiomyomatosis (4), Langerhan’s cell histiocytosis (2), Sickle cell disease (2)	

Descriptive statistics were generated to describe the sample characteristics. To compare the patient characteristics between the fibrotic lung and non-fibrotic lung, we used a t-test for continuous variables and Chi-squared test for categorical variables. Nevertheless, when we compared the proportion of pathology proven cancer between nodules occurring in fibrosis lung and nodules occurring in the non-fibrotic lung for a given range of nodule size, we used Fisher’s exact test. While the exact method is more conservative (compared to Chi-squared test), due to the small sample size for these subgroup analyses, it is a preferable analytic approach as it avoids the potential issue of large sample approximation used in the Chi-squared test. We also used Fisher’s exact test to compare the proportion of each specific type of cancer between fibrosis and non-fibrosis lung for a similar reason. We declared findings as statistically significant if the corresponding p-values were no greater than 0.05. The analysis was performed using SPSS 26.0.

## Results

### Overall distribution of CT scans

**Fig. 1:**
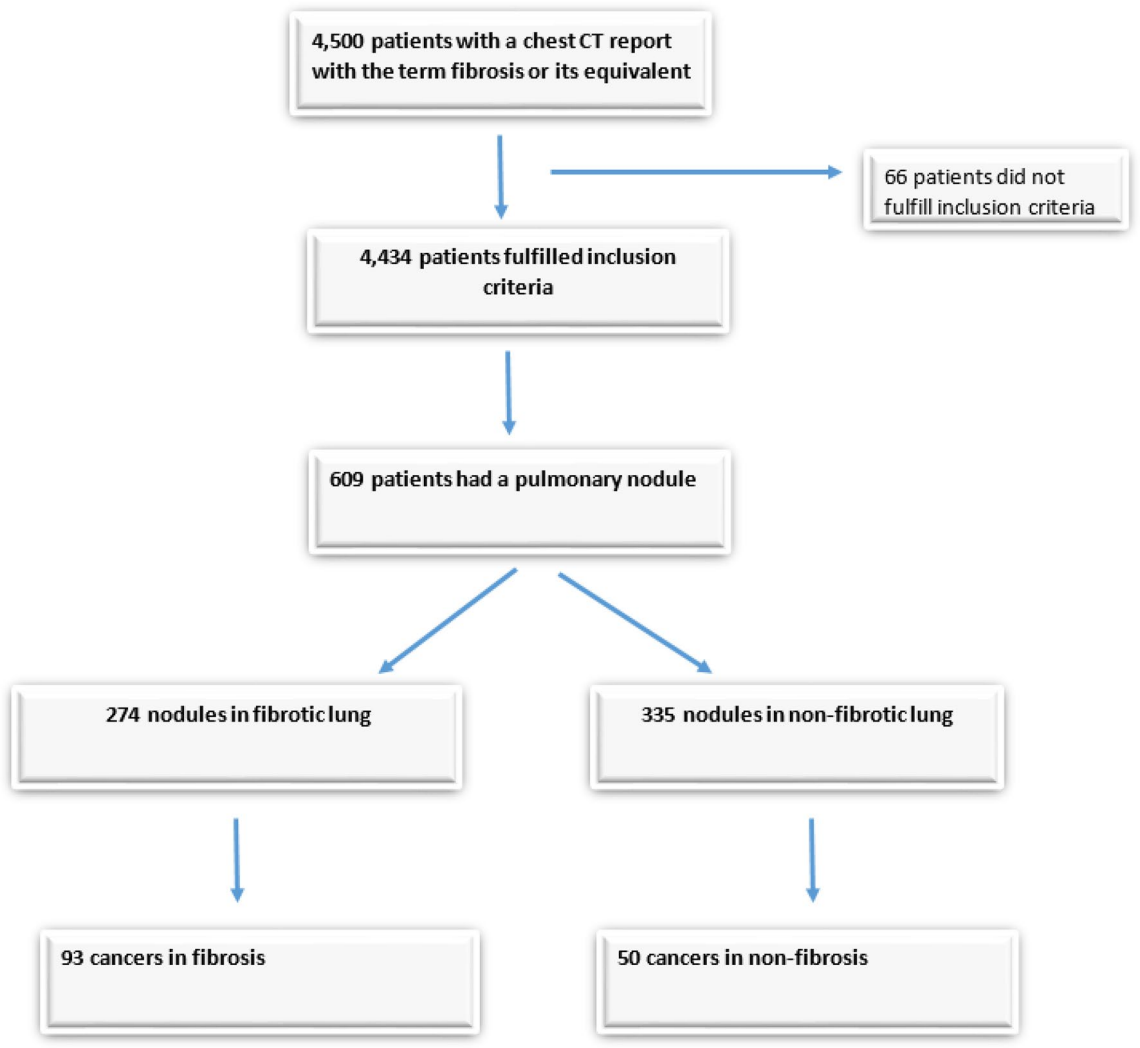
Flowchart

4500 patients with the word fibrosis in their chest CT report were identified. Sixty-six patients did not meet inclusion criteria (age less than 21 at time of first CT scans or images not retrievable) leaving 4434 patients as the subjects for possible nodule analysis (Fig. 1). 3,377 CT scans had pulmonary fibrosis with the most common patterns; airway centered fibrosis (ACF) (n = 952), usual interstitial pneumonitis (UIP) (n = 697), sarcoidosis (n = 516) and nonspecific interstitial pneumonitis (NSIP) (n = 476). Non-fibrotic CT scan diagnoses included bilateral transplant (n = 142), bronchiectasis (n = 63) and emphysema (n = 56) (Table 1).

### Nodules

Six hundred and nine patients were found to have at least one pulmonary nodule on either the first (prevalent lung nodule) or the second CT scan. 274 of the largest pulmonary were in the fibrotic tissue and 335 of the largest pulmonary nodules were in the non-fibrotic lung parenchyma. Patients with the largest lung nodules in fibrosis were slightly older (70 vs 66 years old, p < 0.01). Nodules in fibrosis were on average, larger in size on the first CT than nodules in non-fibrotic lung tissue and grew more by the second CT (Table 2). Nodules occurring in fibrosis were more frequent in the lower lungs than nodules in the non-fibrotic lung. 217 patients with fibrotic nodules and 263 patients with non-fibrotic nodules had follow-up scans allowing doubling time calculation. Non-fibrotic nodules were more likely to remain stable over time or decrease in size, in contrast, fibrotic nodules had a greater propensity to increase in size on follow-up exam (Table 2). Nodules that were not biopsied were considered likely benign if they decreased in size, and were stable for 1 year or more, the patient did not develop symptoms of lung cancer for at least 1 year after the CT or the nodule size was ≤ 6 mm (Table 3).

**Table 2:** Characteristics of fibrotic versus non-fibrotic lung nodules

	Description	Fibrotic lung nodule (N = 274)	Non-fibrotic lung nodule (N = 335)	*p-value
Demographics	Age at first CT	70 (22–95)	66 (25–93)	< 0.01
	Gender (male)	137/274 (50%)	156/335 (47%)	0.50
Nodule characteristics	Location	RUL (77) 28% RML (15) 6% RLL (72) 26% LUL (56) 20% LLL (54) 20%	RUL (83) 25% RML (43) 13% RLL (59) 18% LUL (79) 24% LLL (71) 21%	< 0.01
	Average size(mm) first CT Average size of non-cancer nodules Average size of cancer nodules	16 mm (0–87 mm) 15 mm (range 3–81 mm) 26 mm (range 2–87 mm)	10 mm (0–106 mm) 10 mm (range 2–52 mm) 29 mm (range 2–106 mm)	< 0.05
	Average size(mm) second CT	23 mm (0–167 mm)	11 mm (0–92 mm)	< 0.01
	Two CT scans	217/274 (79%)	263/335 (79%)	0.84
	No change in size	78/217 (36%)	167/263 (63%)	< 0.01
	Decrease in size	15/217 (7%)	25/263 (10%)	< 0.01
	Increase in size	97/217 (45%)	48/263 (18%)	< 0.01
	New nodule	27/217 (12%)	23/263 (9%)	0.18
Lung cancer	Pathology proven cancer	93/274 (34%)	50/335 (15%)	< 0.01
	Squamous cell carcinoma	30/93 (32%)	7/50(14%)	0.02
	Adenocarcinoma	36/93 (39%)	30/50 (60%)	0.13
	Small cell lung cancer	15/93 (16%)	5/50 (10%)	0.31
	Other cancer	12/93 (13%)	8/50 (16%)	0.05

^*^p-values of Chi-squared test for categorical variables and t-test for continuous variables

**Table 3:** Outcomes for pulmonary nodules in the fibrotic and nonfibrotic lung

	Fibrotic nodules	Nonfibrotic nodules
Cancer with biopsy	93	50
Benign		
Benign biopsy	16	14
Decrease in size	10	19
CT stability ≥ 12 months	50	101
Clinical stability > 12 months	55	73
Nodule < 6 mm in size	22	64
Indeterminate		
Suspicious nodule not biopsied	21	8
History of cancer suspicious for metastases	7	6
Total	274	335

### Cancer

**Fig. 2:**
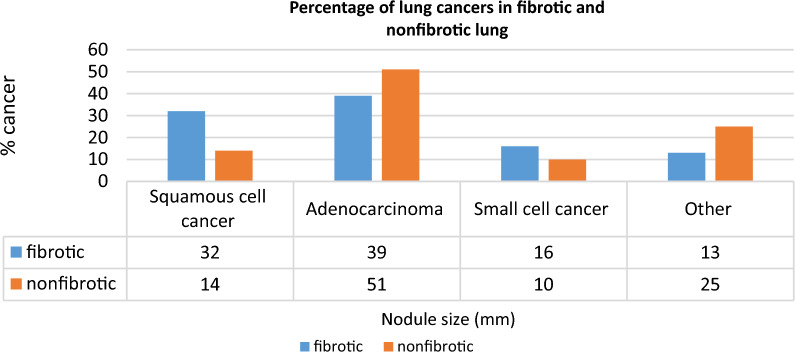
Percentage of cancers occurring in the fibrotic and non-fibrotic lung

Pathology proven cancer was more common in nodules occurring in fibrosis compared to nodules occurring in the non-fibrotic lung (34% vs 15%, p < 0.01). Adenocarcinoma was the most common cell type in both groups but more frequent in cancers occurring in non-fibrotic tissue. Squamous cell cancer represented 32% of cancers occurring in fibrotic tissue (p = 0.02) (Fig. 2). Cancers occurred more frequently in association with UIP, combined pulmonary fibrosis and emphysema (CPFE), and unilateral lung transplant pattern and were less common with NSIP, ACF, and sarcoid (Table 4). Larger lung nodules were more likely to be cancer than smaller nodules in fibrotic and non-fibrotic lungs. In the non-fibrotic lung, 1 of 126 (0.8%) of nodules measuring 1 to 6 mm were cancer. In contrast, the cancerous nodules in fibrosis were smaller with 5 of 49 (10.2%) nodules measuring 1 to 6 mm representing biopsy-proven cancer (p < 0.01) (Table 5).

**Table 4:** Fibrosis type and its association with cancer

Type of fibrosis (number of patients)	# with cancer	Percentage with cancer
Asbestosis (N = 8)	2	25.0
Unclassifiable (N = 8)	2	25.0
Radiation (N = 24)	3	12.5
Vasculitis (N = 9)	1	11.1
Combined fibrosis and emphysema (N = 113)	9	8.0
Usual interstitial pneumonitis (N = 697)	43	6.2
Unilateral transplant (N = 244)	12	4.9
Nonspecific interstitial pneumonitis (N = 476)	7	1.5
Airway centered fibrosis (N = 952)	10	1.1
UIP associated with CTD (N = 109)	1	0.9
Sarcoid (N = 516)	3	0.6

**Table 5:** Percentage of patients with cancer based on the size of the nodule

Nodule size	# in non-fibrosis	# (%) cancer	# in fibrosis	# (%) cancer	*p-value
1–6 mm	126	1 (0.8%)	49	5 (10.2%)	< 0.01
7–12 mm	110	11 (10.0%)	81	19 (23.5%)	0.02
13–20 mm	50	12 (24.0%)	57	17 (29.8%)	0.52
21–30 mm	19	7 (36.8%)	37	24 (64.9%)	0.05
31–40 mm	10	4 (40.0%)	21	10 (47.6%)	0.99
41–50 mm	11	8 (72.7%)	16	10 (62.5%)	0.69
> 50 mm	9	7 (77.8%)	13	8 (61.5%)	0.65
Total	335	51 (15.2%)	274	93(33.9%)	< 0.01

^*^p-values for Fisher’s exact test

The doubling time for squamous cell cancer was shorter in the fibrotic lung compared to non-fibrotic lung, however, the difference was not statistically significant (105 days vs 179 days, p = 0.24). Adenocarcinoma in the fibrotic and non-fibrotic lung had similar doubling times (235 days vs 194 days, p = 0.24). The same was true for small cell cancer with doubling times of 43 days and 28 days (p = 0.23). When considering only squamous cell cancer and adenocarcinomas in the fibrotic and non-fibrotic lung, the largest percentage of nodules in the group with doubling times of < 90 days were squamous cell cancer and adenocarcinoma in fibrosis. Adenocarcinoma in the non-fibrotic lung had the longest doubling times of > 360 days (Table 6).

**Table 6:** Doubling times of nodules in the fibrotic and non-fibrotic lung

	Fibrotic lung	Non-fibrotic lung	*p-value
Squamous cell carcinoma	105 days (range 17–321) N = 22	179 days (range 95–263) N = 2	0.24
Adenocarcinoma	235 days (range 20–1001) N = 24	194 days (range 34–860) N = 10	0.24
Small cell cancer	43 days (range 21–95) N = 6	28 days (range 22–33) N = 2	0.23
Metastases	None	113 days (range 25–290) N = 4	NA
Other	76 days (range 12–225) N = 7	162 days (range 41–283) N = 2	0.11

^*^p-values for t-test

16 new nodules were identified on the second CT obtained ≤ 18 months after the first CT scan in non-fibrotic lung and 2 (13%) were cancer; 1 adenocarcinoma and 1 squamous cell cancer. 15 incident lung nodules were found in fibrotic lung and 8 (53%) were diagnosed as cancer; 2 were adenocarcinoma, 2 were small cell cancer, and 3 were squamous cell cancer (p = 0.02). (Table 7).

**Table 7:** Association of incident lung nodules (Occurring ≤ 18 months after prior CT) and cancer in the fibrotic and non-fibrotic lung

	Fibrosis (N = 15)	Non-fibrosis (N = 16)	*p-value
Cancer	8 (53.3%)	2 (12.5%)	0.02
Adenocarcinoma	2 (13.3%)	1 (6.3%)	0.60
Squamous cell cancer	3 (20.0%)	1 (6.3%)	0.33
Small cell cancer	2 (13.3%)	0 (0.0%)	0.23
Other	1 (6.7%)	0 (0.0%)	0.48
No biopsy	7 (43.3%)	14 (87.5%)	0.02

^*^p-values for Fisher’s exact test

Overall, 42 nodules were deemed suspicious based on their size at initial CT or their doubling time but did not have biopsies. The majority were located in the fibrotic lung (n = 28). 13 of the 42 patients had a known malignancy and nodules were deemed metastatic clinically. 29 were suspicious for primary lung cancer but not biopsied due to the patient clinical condition. 21/29 (72%) suspicious nodules were located in the fibrotic lung. Despite their suspicious appearance, these nodules were not considered cancer unless a biopsy was performed (Table 8).

**Table 8:** Adenocarcinoma and squamous cell cancer doubling times based on location in fibrotic or non-fibrotic lung

	Adenocarcinoma fibrosis (%)	Squamous fibrosis (%)	Adenocarcinoma non-fibrosis (%)	Squamous non-fibrosis (%)	Total (%)
1–90 days	15	16	7	0	38
91–180 days	12	12	6	3	32
181–360 days	7	5	5	2	19
> 361 days	9	0	2	0	11

## Discussion

The Fleischner Society’s 2005 article was most impactful because it stated that not all nodules in the lung are the same and provided guidelines for follow-up of nodules based on the size of the nodule and patients’ risk of cancer [20]. The new guidelines minimized the number of recommended chest CT scans for nodule follow-up. These guidelines have continued to evolve due to the work of many investigators who have pushed the upper limit of a positive result which would require short interval follow-up to prevent excess CT scans while diagnosing cancer at the earliest stage [21]. The Fleischner Society’s 2013 guidelines [22], described unique follow-up intervals for non-solid nodules that do not depend on smoking history but continue for a longer period of time (5 years), and currently, the 2017 guidelines recommend follow-up of nodules < 6 mm at most in 1 year [23]. The American College of Radiology (ACR) Lung-RADS Version 1.1 released in 2019 allows yearly follow-up for high-risk smoking patients with nodules less than 6 mm in size [24]. The International Early Lung Cancer Action Program (I-ELCAP) recommends 6 mm and below as a cut-off for yearly screening [25]. The progressive recommendations are derived from accumulated research on the likelihood that a nodule is cancer and how fast will it grow.

Our research has shown that overall nodules in fibrosis are more likely to be cancer (34% vs 15%, p < 0.01), and nodules that are less than 6 mm had a 5/49 (10.2%) chance of being lung cancer (3 squamous cell cancers and 2 adenocarcinomas). This finding has important implications for work-up; LungRADS recommends yearly follow-up for baseline nodules less than 6 mm because of a < 1% risk of malignancy. A 10.2% risk of cancer for small nodules in fibrosis is comparable to a *LungRADS 4A, probably suspicious risk*, in the smoking population, and would warrant a 3-month follow-up or PET scan [24].

Nodules identified on baseline lung cancer screening are less aggressive than incident lung nodules identified on follow-up exam ≤ 18 months after the first. The I-ELCAP researchers demonstrated 4,959 new nodules on a follow-up exam and 179 (3.6%) were cancer [25]. In contrast, in fibrosis, cancer was identified in 53% of new nodules. This would be equivalent to a *LungRADS 4B, suspicious,* with a greater than 15% risk of cancer and require a PET scan or tissue sampling. An alternative for new nodules is a 1-month follow-up to differentiate early infection [24]. A 1-month follow-up for a new nodule in a patient with pulmonary fibrosis would be the next best step due to the near equal likelihood of an infectious or neoplastic etiology.

Features suggestive of cancer including increasing size of a nodule or new nodules were more common in the fibrotic lung. Benign features including nodule stability or decreasing size were more common in non-fibrotic lung nodules. Volume doubling times are a well-established method to quantify the aggressiveness of lung cancer. Small cell cancers have some of the fastest doubling times and adenocarcinomas are typically slower [26]. Squamous cell cancers with their overall faster doubling times were more common in fibrotic than non-fibrotic lung but adenocarcinoma (39%) remained the most common cell type in the fibrotic lung. Zhang et al. found that lung cancer risk factors did not affect the aggressiveness of lung cancers [27]. Our results are similar, fibrosis is a risk factor for lung cancer yet squamous cell cancer had only a slightly more rapid doubling time in fibrotic versus non-fibrotic lung, adenocarcinoma was not significantly different. Our results complimented Siddique et al. with histology as the predominant driver of tumor doubling time [28].

The risk of cancer was not equally distributed in all types of fibrosis. Patients with asbestosis were at the highest risk but the numbers of patients were small. UIP and CPFE had a 6.2% and 8.0% risk of lung cancer which is remarkably similar to findings reported by Song et al. with a 6.4% prevalence of lung cancer in IPF patients. The risk of lung cancer increased over time ranging from 1.7% at year one of diagnosis to 7.0% by year 5 [29]. New treatments for patients with pulmonary fibrosis increase life expectancy and might be associated with an increased lung cancer diagnosis. Lung cancer in patients with pulmonary fibrosis was associated with increased 5-year mortality [30]. An optimal screening regimen will be necessary for the earliest diagnosis and intervention.

The limitations of our study include the retrospective nature of the research with variable dose and slice thickness. The exams were read by a single reader who also reviewed the report to make sure the largest nodule was included. Many patients with fibrosis are too sick to have a biopsy despite the suspicious morphology of a nodule and its rapid doubling times. If more biopsies had been performed, the number of cancers diagnosed would likely be significantly higher in the fibrotic lung; many nodules were suspicious based on doubling time calculations, especially in the fibrotic lung.

## Conclusions

Nodules in fibrotic lung are more likely to be cancer than nodules in non-fibrotic lung. Squamous cell cancer, with its shorter doubling times, occurs more frequently in the fibrotic lung but adenocarcinoma remains the dominant cancer type. Nodules less than 6 mm in size are more likely to be lung cancer in the fibrotic lung than in the non-fibrotic lung and should be followed closely.

The goal of a successful screening program is to minimize false-positive exams while diagnosing cancer as early as possible. There is a tradeoff between the two; decreasing false-positive exams requires setting the threshold higher and allowing cancers to be larger at the time of diagnosis. The acceptable risk of malignancy is less than 1% providing the rationale for Lung-Rads 1-year follow-up interval for solid non-calcified nodules less than 6 mm in size. The results of our research suggest the possible need for modifications to the screening regimen in patients with fibrosis. Prospective studies will be necessary to confirm these findings.

## Data Availability

De-identified data is available upon request for review.

## Abbreviations

UIP: Usual interstitial pneumonia
ACF: Airway-centered fibrosis
NSIP: Nonspecific interstitial pneumonitis
CPFE: Combined pulmonary fibrosis and emphysema
IPF: Idiopathic pulmonary fibrosis
ACR: American College of Radiology
CTD: Connective tissue disease
ILD: Interstitial lung disease
HU: Hounsfield Unit
PA: Pulmonary artery

## References

[1] Yankelevitz DF, Smith JP (2013). Understanding the core result of the National Lung Screening Trial. N Engl J Med.

[2] Aberle DR (2011). Reduced lung-cancer mortality with low-dose computed tomographic screening. N Engl J Med.

[3] Henschke CI, McCauley DI, Yankelevitz DF, Naidich DP, McGuinness G, Miettinen OS, Libby DM, Pasmantier MW, Koizumi J, Altorki NK, Smith JP (1999). Early lung cancer action project: overall design and findings from baseline screening. Lancet.

[4] Yankelevitz D, Henschke C (2016). Low-dose CT screening—determining the right interval. Nat Rev Clin Oncol.

[5] Henschke CI (2012). Lung cancers diagnosed at annual CT screening: volume-doubling times. Radiology.

[6] Naccache JM, Gibiot Q, Monnet I (2018). Lung cancer and interstitial lung disease: a literature review. J Thorac Dis.

[7] Yoon JH, Nouraie M, Chen X, Zou RH, Sellares J, Veraldi KL, Chiarchiaro J, Lindell K, Wilson DO, Kaminski N, Burns T, Trejo Bittar H, Yousem S, Gibson K, Kass DJ (2018). Characteristics of lung cancer among patients with idiopathic pulmonary fibrosis and interstitial lung disease—analysis of institutional and population data. Respir Res.

[8] Ozawa Y, Suda T, Naito T, Enomoto N, Hashimoto D, Fujisawa T (2009). Cumulative incidence of and predictive factors for lung cancer in IPF. Respirology.

[9] Jung HI, Park JS, Lee MY, Park B, Kim HJ, Park SH, Choi WI, Lee CW (2018). Prevalence of lung cancer in patients with interstitial lung disease is higher than in those with chronic obstructive pulmonary disease. Medicine (Baltimore).

[10] Whittaker Brown SA, Padilla M, Mhango G, Powell C, Salvatore M, Henschke C, Yankelevitz D, Sigel K, de Torres JP, Wisnivesky J (2019). Interstitial lung abnormalities and lung cancer risk in the National Lung Screening trial. Chest.

[11] Murthy S, Larson-Casey JL, Ryan AJ (2015). Alternative activation of macrophages and pulmonary fibrosis are modulated by scavenger receptor, macrophage receptor with collagenous structure. FASEB J.

[12] Xu F, Wei Y, Tang Z, Liu B, Dong J (2020). Tumor associated macrophages in lung cancer: friend or foe? (Review). Mol Med Rep.

[13] US Preventive Services Task Force (2021). Screening for lung cancer: US preventive services task force recommendation statement. JAMA.

[14] Wang Y, Midthun DE, Wampfler JA, Deng B, Stoddard SM, Zhang S, Yang P (2015). Trends in the proportion of patients with lung cancer meeting screening criteria. JAMA.

[15] Jin J (2021). Screening for lung cancer. JAMA.

[16] Kwak N, Park CM, Lee J, Park YS, Lee SM, Yim JJ, Yoo CG, Kim YW, Han SK, Lee CH (2014). Lung cancer risk among patients with combined pulmonary fibrosis and emphysema. Respir Med.

[17] Schwartz LH, Litière S, de Vries E (2016). RECIST 1.1-update and clarification: from the RECIST committee. Eur J Cancer.

[18] Mehrara E, Forssell-Aronsson E, Ahlman H, Bernhardt P (2007). Specific growth rate versus doubling time for quantitative characterization of tumor growth rate. Cancer Res.

[19] http://radclass.mudr.org/content/doubling-time-calculation-growth-rate-lesion-or-mass.

[20] MacMahon H, Austin JH, Gamsu G, Herold CJ, Jett JR, Naidich DP, Patz EF, Swensen SJ, Fleischner Society (2005). Guidelines for management of small pulmonary nodules detected on CT scans: a statement from the Fleischner Society. Radiology.

[21] Yip R, Henschke CI, Yankelevitz DF, Smith JP (2014). CT screening for lung cancer: alternative definitions of positive test result based on the national lung screening trial and international early lung cancer action program databases. Radiology.

[22] Naidich DP, Bankier AA, MacMahon H (2013). Recommendations for the management of subsolid pulmonary nodules detected at CT: a statement from the Fleischner Society. Radiology.

[23] MacMahon H, Naidich DP, Goo JM, Lee KS, Leung ANC, Mayo JR, Mehta AC, Ohno Y, Powell CA, Prokop M, Rubin GD, Schaefer-Prokop CM, Travis WD, Van Schil PE, Bankier AA (2017). Guidelines for management of incidental pulmonary nodules detected on CT images: from the Fleischner Society 2017. Radiology.

[24] Kastner J, Hossain R, Jeudy J, Dako F, Mehta V, Dalal S, Dharaiya E, White C (2021). Lung-RADS version 1.0 versus lung-RADS version 1.1: comparison of categories using nodules from the national lung screening trial. Radiology.

[25] Henschke CI, Yip R, Yankelevitz DF, Smith JP (2013). Definition of a positive test result in computed tomography screening for lung cancer: a cohort study. Ann Intern Med.

[26] Henschke CI, Salvatore M, Cham M, Powell CA, DiFabrizio L, Flores R, Kaufman A, Eber C, Yip R, Yankelevitz DF, International Early Lung Cancer Action Program Investigators (2018). Baseline and annual repeat rounds of screening: implications for optimal regimens of screening. Eur Radiol.

[27] Zhang L, Yip R, Jirapatnakul A, Li M, Cai Q, Henschke CI, Yankelevitz DF, I-ELCAP Investigators (2020). Lung cancer screening intervals based on cancer risk. Lung Cancer.

[28] Siddique M, Yip R, Henschke CI, Yankelevitz DF (2021). PET standardized uptake values of primary lung cancer for comparison with tumor volume doubling times. Clin Imaging.

[29] Song MJ, Kim SY, Park MS, Kang MJ, Lee SH, Park SC (2021). A nationwide population-based study of incidence and mortality of lung cancer in idiopathic pulmonary fibrosis. Sci Rep.

[30] Kato E, Takayanagi N, Takaku Y, Kagiyama N, Kanauchi T, Ishiguro T, Sugita Y (2018). Incidence and predictive factors of lung cancer in patients with idiopathic pulmonary fibrosis. ERJ Open Res.

